# Rationale and design of a randomized, controlled multicentre clinical trial to evaluate the effect of bromocriptine on left ventricular function in women with peripartum cardiomyopathy

**DOI:** 10.1007/s00392-015-0869-5

**Published:** 2015-05-31

**Authors:** Arash Haghikia, Edith Podewski, Dominik Berliner, Kristina Sonnenschein, Dieter Fischer, Christiane E. Angermann, Michael Böhm, Philipp Röntgen, Johann Bauersachs, Denise Hilfiker-Kleiner

**Affiliations:** Department of Cardiology and Angiology, Hannover Medical School, Carl-Neuberg-Str. 1, 30625 Hannover, Germany; Department of Cardiology and Angiology, University Hospital Muenster, Muenster, Germany; Department of Internal Medicine I, Comprehensive Heart Failure Center, University Hospital of Wuerzburg, Würzburg, Germany; Department of Internal Medicine III, University Hospital of Saarland, Homburg/Saar, Germany

**Keywords:** Peripartum cardiomyopathy, Bromocriptine, Prolactin, Heart failure

## Abstract

**Background:**

Peripartum cardiomyopathy (PPCM) is an idiopathic heart disease that develops in the last month of pregnancy and/or the first months following delivery in previously healthy women and may lead to acute heart failure. A cleaved fragment of the nursing hormone prolactin is considered essential in the pathophysiology of PPCM. To date, no specific therapy has been tested for PPCM in a randomized controlled trial of adequate size.

**Aims:**

The purpose of this trial is to investigate the safety of the dopamin-D2-receptor agonist bromocriptine and its effects on left ventricular (LV) function in women with PPCM.

**Methods:**

This is an 11 center German trial with a prospective randomized controlled open-label design. The trial enrolls females with newly diagnosed PPCM according to European Society of Cardiology criteria with a LV ejection fraction (LVEF) <35 %. Patients are randomized 1:1 to either best supportive care (BSC) including standard heart failure therapy plus 8 weeks of bromocriptine therapy (2.5 mg b.i.d. for 14 days and 2.5 mg q.d. from day 15 to 56) or to BSC plus 1 week of low-dose bromocriptine (2.5 mg q.d.) with anticoagulant therapy at a prophylactic dose administered during the period of bromocriptine treatment in both groups. The primary endpoint is change in LVEF from baseline to 6 months follow-up as assessed by cardiac magnetic resonance imaging (or echocardiography if CMR is not tolerated). The secondary endpoints are hospitalization for worsening heart failure, heart transplantation, and all-cause mortality during follow-up or a combination of these endpoints. A total of 60 patients will be recruited (including 6 potential dropouts) giving a power of 0.9 for an expected LVEF change of 10.8 % between treatment groups at 6 months.

**Perspective:**

This trial will provide important knowledge on potential benefits and safety of prolonged inhibition of prolactin release with bromocriptine in addition to standard heart failure therapy in newly diagnosed PPCM.

Trial registration: ClinicalTrials.gov Identifier: NCT00998556.

## Introduction

Peripartum cardiomyopathy (PPCM) is an idiopathic cardiomyopathy toward the end of pregnancy or in the months following delivery presenting with acute heart failure with reduced left ventricular (LV) function [1, 2]. The incidence of PPCM ranges from 1:299 births in Haiti to about 1:1000 in South Africa and in the USA [2, 3].

Moreover, recent studies suggest a growing incidence of PPCM [3] which may be partly due to certain socio-environmental factors that still need to be identified and partly due to improved awareness to this disease and advances in diagnostic opportunities.

Although PPCM is presumed to be associated with a higher likelihood of recovery of LV function than other cardiomyopathies [4], we recently reported on 15 % treatment failure in a German registry with prospective data of 96 PPCM patients despite optimal medical therapy.

The disease is potentially life threatening with regionally varying mortality rates ranging between 2 % in our registry under contemporary management concepts, and up to 50 % in other studies [5–7].

Importantly, a number of studies on PPCM cohorts of different ethnicities have demonstrated that the presence of severe LV dysfunction at baseline is associated with a low probability of full cardiac recovery [5, 8]. Among the triggering factors the nursing hormone prolactin is considered to play a key role in the pathophysiology of (PPCM). Under circumstances of enhanced oxidative stress that typically occurs toward the end of pregnancy and during delivery, prolactin is increasingly cleaved into a 16 kDa fragment (16 kDa PRL) under conditions of defective antioxidative mechanisms [9]. The 16 kDa PRL is known to exert detrimental effects on the microvasculature of the heart leading to cardiac injury and dysfunction [9, 10].

Despite these advances in understanding the pathomechanisms of PPCM, clinical trials testing disease-specific therapeutics still remain scarce. In fact, apart from standard heart failure medication no specific therapy is available for PPCM to date.

Thus, there is a need for new therapeutic strategies to counteract the detrimental pathomechanisms in PPCM and enhance myocardial recovery. Given the key role of prolactin in the pathogenesis of PPCM, prolonged inhibition of prolactin release by the dopamin-D2-receptor agonist bromocriptine may represent one therapeutic opportunity. After this approach had been successfully tested in single cases [11], a prospective, single-center, randomized, open-label, pilot study of African women with newly diagnosed PPCM was performed which compared standard heart failure care in PPCM patients versus standard care plus treatment with bromocriptine for 8 weeks with 10 patients per group (Table 1) [12].

Patients receiving bromocriptine displayed greater recovery of ejection fraction at 6 months and were less likely to experience the composite end point of poor outcome defined as death, New York Heart Association (NYHA) functional class III/IV, or left ventricular ejection fraction <35 % at 6 months than the patients receiving standard care. One patient in the bromocriptine group died compared with 4 patients in the standard care group. Although of limited small size, this trial justified the concept of targeting prolactin as a novel therapeutic strategy for PPCM and provided the rationale for a larger randomized and controlled clinical trial with bromocriptine (Table 1). This concept was further supported by the results of the German PPCM registry [5] demonstrating cardiac improvement in 96 % of the patients receiving bromocriptine in a non-randomized manner in addition to a beta-blocker and angiotensin-converting enzyme (ACE-) inhibitors (Table 1).

**Table 1:** Summary of published studies investigating bromocriptine in patients with PPCM

Study	Sample size (*n*)	Treatment duration	Randomized?	Controlled?	Endpoint
Sliwa et al. [12]	20	8 weeks	Yes	Yes	LVEF at 6-month follow-up
Haghikia et al. [5]	96	4 weeks	No	No	LVEF at 6-month follow-up

Thus, the objective of the present study is to determine in a randomized and controlled design and with adequate sample size the therapeutic potential and safety of bromocriptine in patients with acute heart failure and reduced LV function due to newly diagnosed PPCM.

## Study design

**Fig. 1:**
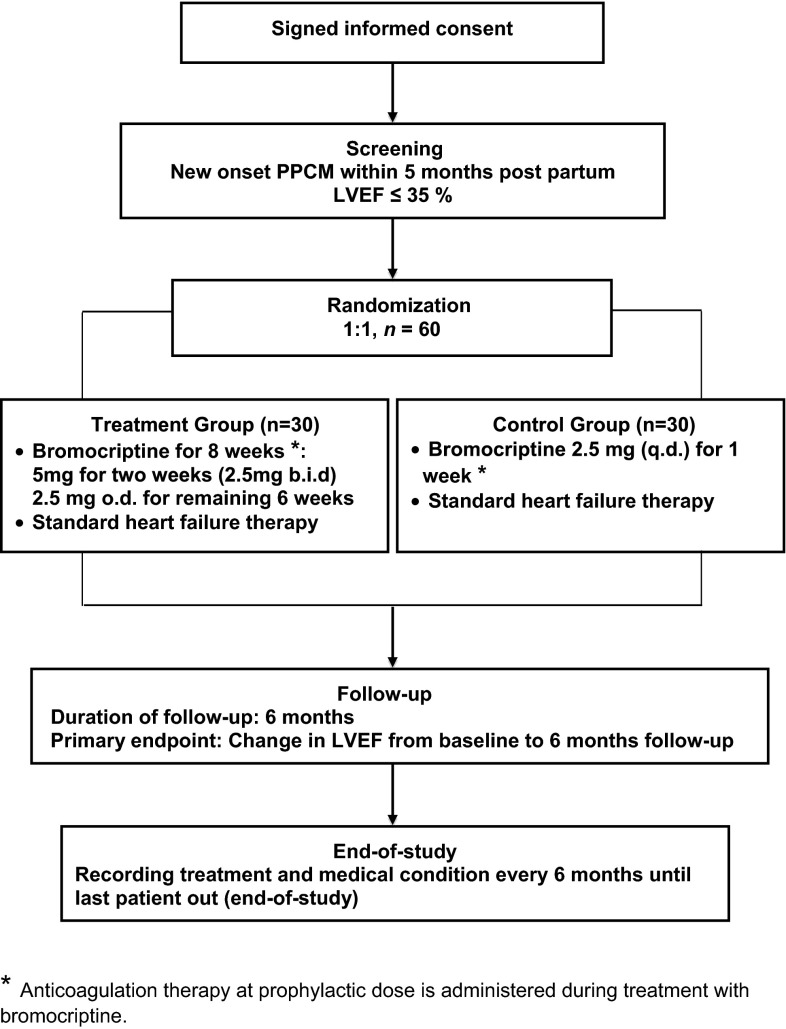
Study design. * Asterisks* indicates anticoagulation therapy at prophylactic dose is administered during treatment with bromocriptine.

This study is a prospective, randomized, controlled trial, which is conducted in 11 centres in Germany (Fig. 1).

## Eligibility

The inclusion and exclusion criteria are summarized in Table 2. The aim is to enroll patients with newly diagnosed PPCM in accordance to the definition proposed by the Working Group on PPCM from the Heart Failure Association of the European Society of Cardiology (ESC) [1] which is as follows: (a) evidence of heart failure secondary to systolic dysfunction toward the end of pregnancy or in the months following delivery, where no other cause of heart failure is found; (b) it is a diagnosis of exclusion; (c) the left ventricle may not be dilated but the ejection fraction is nearly always reduced below 45 %.

**Table 2:** Summarized inclusion and exclusion criteria

Inclusion criteria	Exclusion criteria
Confirmed PPCM according to ESC definition Time-window within 5 months postpartum LVEF ≤35 % At least 18 years of age Ability to give written informed consent	Preexisting cardiac disease (except PPCM with complete resolution) Any preexisting serious conditions Previous cardiac surgery or percutaneous coronary intervention History of alcohol and/or any other drug abuse Contraindication to the planned therapy Concomitant therapy other than specified in the trial protocol Expected low compliance (e.g., by travel distance to trial site) Concomitant participation in other clinical trials

*ESC* European Society of Cardiology, *PPCM* peripartum cardiomyopathy, *LVEF* left ventricular ejection fraction

Specific inclusion criteria include (1) the confirmed diagnosis of new onset PPCM within a time-window of 5 months postpartum; (2) LV ejection fraction (LVEF) below 35 % as assessed by echocardiography; (3) the age of 18 years or older; and (4) the ability to give written informed consent. PPCM is defined as a non-familial form of peripartum heart failure characterized as an “idiopathic cardiomyopathy presenting with heart failure secondary to left ventricular (LV) systolic dysfunction towards the end of pregnancy or in the months following delivery, where no other cause of heart failure is found”.

## Registration, screening, enrolment, and randomization

### Registration and screening

Potential participants are all women with PPCM in the first 5 months postpartum displaying clinical signs of heart failure. After explanation of the nature and scope of the study and a sufficient period of reflection, the investigator must obtain the informed consent by means of a standard written statement in non-technical language. Patients may withdraw their consent of participation at any time of the trial without providing a reason. Patients with heart failure symptoms are screened for PPCM by echocardiography and further inclusion and exclusion criteria are evaluated during the enrolment and randomization visit (visit 1).

### Enrolment and randomization (visit 1)

At visit 1, patient eligibility is evaluated based on the inclusion/exclusion criteria (Table 2). If the patient is considered eligible, the medical history is assessed, physical examination, ECG, and echocardiography are performed and the NYHA class and vital signs are documented. The baseline LVEF has to be determined by cardiac magnetic resonance imaging (CMR) within 5 days after beginning of bromocriptine therapy. Alternatively, if the patient does not tolerate CMR or CMR is temporarily not available, it may as an exception be replaced by two separate echocardiograms with determination of the LVEFs (Simpson’s method) by two independent investigators blinded to each others results. Moreover, laboratory tests for NTproBNP, hemoglobin, serum sodium and potassium, glucose, aspartate aminotransferase, alanine aminotransferase, total bilirubin, blood urea, nitrogen, and serum creatinine are measured. The quality of life is assessed by means of a questionnaire.

Patients who are not eligible to participate in the study are asked to provide their demographic (age, parity, ethnic background) and clinical data for the German PPCM registry [5] and the international registry on PPCM, which is part of the ESC EURObservational Research Programme (http://www.eorp.org) [13].

A central computerized randomization (modified according to Pocock 15 and Pocock and Simon 16) of patients fulfilling the inclusion criteria is performed at the Coordination Centre for Clinical Trials at the University of Leipzig (ZKS Leipzig KKS). Patients are randomized in a 1:1 ratio to either the treatment group or the control group. The treatment group receives bromocriptine therapy at a dose of 2.5 mg b.i.d for the first 2 weeks and 2.5 mg o.d. for another 6 weeks (total treatment of 8 weeks with bromocriptine). The control group receives low-dose bromocriptine (2.5 mg o.d.) for up to 1 week to stop lactation. In both groups anticoagulant therapy at a prophylactic dose is administered during the period of bromocriptine treatment to prevent thrombotic side effects of bromocriptine.

Both groups are additionally treated with standard heart failure medication as per current guidelines [14–17]. Specifically, this therapy includes ACE inhibitors or Angiotensin II receptor blockers (ARBs), beta-blockers, mineralocorticoid receptor blockers (MRAs), diuretics, and ivabradine if indicated. In addition, prolactin, NT-pro-BNP, and a set of additional biomarkers for cardiovascular disease, inflammation, and markers specific for the pathophysiology of PPCM will be measured at each visit. Additionally, novel potential biomarkers derived from experimental studies in ongoing PPCM research in our and other labs will be tested for diagnostic accuracy and prognostic value.

### Monitoring of safety and tolerability and follow-up (visits 2–4)

After enrolment, 3 study visits take place during a follow-up period of 6 months: Visit 2 at week 2, visit 3 at week 4, and visit 4 at the end of week 8. At each visit the medical history and quality of life are assessed, and adverse events (AEs) and serious adverse events (SAE) are documented. Further, NYHA class and vital signs are documented and physical examination, ECG, echocardiography, and laboratory tests are performed. The compliance to study medication is assessed and the standard heart failure medication is adjusted if necessary.

### End of follow-up at month 6 (visit 5)

At visit 5 the 6 months LVEF is determined by CMR. This visit also includes all procedures performed at the previous visits. The end of study is specified as the visit 5 of the last patient. Until then the treatment and medical conditions of the other patients are recorded every 6 months.

## Study objectives

### Primary objectives

The purpose of the study is to evaluate the effect of prolonged blockade of prolactin with bromocriptine on left ventricular function in women with PPCM. The primary endpoint is the change of LVEF from baseline to 6 months follow-up determined at by CMR (Table 3).

### Secondary objectives

The secondary endpoints are rate of hospitalizations for worsening heart failure, heart transplantation, and mortality during 6 months follow-up and the combination of the afore mentioned endpoints (Table 3).

**Table 3:** Pre-specified endpoints

Primary endpoint	Secondary endpoints
Change in LVEF from baseline to 6 months follow-up	Hospitalization for heart failure symptoms during 6 months follow-up Necessity of heart transplantation during 6 months follow-up Mortality during 6 months follow-up Combination of hospitalization, heart transplantation, and mortality during 6 months follow-up

### Statistical consideration

The estimated average change of LVEF during 6 months for the primary endpoint was based on the previous pilot study including 10 patients with and 10 patients without bromocriptine where the average change of LVEF during 6 months was by absolute 23 % higher in the group with bromocriptine [12]. We assume that the average difference between the groups in this study is 12 % absolute, corresponding to a 52 % relative reduction of the effect seen in the pilot study. If we assume that the patients in the control group who receive 1/10 of the cumulative dose given to the patients of the treatment group might have 1/10 of the benefit, this will reduce the average gain in LVEF to 10.8 % in absolute terms with a standard variation of 12 %. Consequently, to detect this difference in a two-sided difference test with a type I error level of 0.05 and a power of 0.9, a total of 54 patients is needed, 27 per group. Therefore, a total of 60 patients (30 per group) should be sufficient to allow for 3 dropouts per group. Regarding the primary endpoint, an analysis of covariance will be performed. The follow-up LVEF will be defined as dependent variable, the treatment group as fixed factor and the baseline LVEF as covariate. As for the secondary endpoints, frequency tables of rates with appropriate 95 % confidence intervals will be performed and comparisons using contingency tables will be compiled. If sufficient numbers of events occur, additional time-to-event analyses, such as Kaplan–Meier estimates of survival curves, will be carried out.

A final detailed plan for the statistical analyses will be developed prior to the end of study and the start of the final analysis.

### Study duration, interim analyses, and early termination

The expected total duration of this study is 60 months. The estimated start of data cleaning is scheduled for July, 2016. Given the rather small sample size, there will be no interim analysis. Therefore, early termination due to putative statistically significant interim results will not occur.

### Current status

To date, the protocol was approved by the Ethics Committee and the competent federal authority (BfArm). The study is being conducted in accordance with the Declaration of Helsinki (Version Somerset West 1996), German laws and the ICH guidelines for Good Clinical Practice (GCP). The trial has been registered on ClinicalTrials.gov, NCT00998556. Patients started enrolling in this trial on 30 June 2010. As of October 2014, 108 patients have been screened in 10 trial centres, of whom 49 patients have been enrolled. Of the remaining 59 patients, 10 patients have denied study participation, and 49 patients did not meet the inclusion criteria. So far, 6 patients have dropped out after randomization, of whom 5 patients were randomized to the control group but wished to receive the bromocriptine therapy according to the treatment, and one patient lost contact to the study centre. Although the analysis of the primary endpoint will be performed according to the intention-to-treat principle, additional per protocol analyses are also planned which will not be used for hypothesis calculations but will serve to estimate and discuss potential biases of the intention-to-treat analysis.

### Safety considerations

Bromocriptine is a dopamine receptor agonist, available on the market for many years, and indicated for treatment of hyperprolactinemia-associated dysfunctions, acromegaly, and Parkinson’s disease. In the postpartum phase, bromocriptine has been used worldwide since 1980 to suppress lactation. The side effects of bromocriptine are well-known [18] and include nausea, headache, dizziness, fatigue, lightheadedness, vomiting, abdominal cramps, nasal congestion, constipation, diarrhea, and drowsiness. Some concerns have been raised about a potential risk for cerebral and cardiovascular complications, as emphasized in some case reports describing stroke and coronary artery thrombosis [19, 20], features that may be more associated with the use of higher dose of bromocriptine. In our study, we use low-dose bromocriptine of 2.5–5 mg/day and specific attention is paid to ensure sufficient prophylactic anticoagulant therapy during treatment with bromocriptine as the procoagulatory activity in the peripartum phase appears to be increased [21]. Recently a review of the available data on safety and effectiveness of bromocriptine in controlling breast milk production after childbirth by the Coordination Group for Mutual Recognition and Decentralised Procedures—Human (CMDh) led to the conclusion that bromocriptine should not be used routinely for preventing or stopping milk production, and must not be used in women at increased risk of serious side effects including women with various disorders that increase blood pressure or who have or have had heart disease or severe psychiatric disorders. Following this recommendation, the European Medicines Agency (EMA) endorsed the restricted use of bromocriptine for stopping milk production on August 21st 2014.

However, this endorsement does not reflect the findings of the German PPCM registry [5], the ESC PPCM registry [13] or the safety data of this trial so far. The data of the PPCM registry showed that almost all patients, 96 % (55/57), who underwent a treatment combination of beta-blockers, ACE inhibitors/angiotensin receptor blockers, and bromocriptine under prophylactic anticoagulation improved their condition without any incidence of bromocriptine-associated side effects In particular, no abnormally high blood pressures or psychiatric disorders were observed. The ESC PPCM registry has already enrolled over 200 cases, of whom 75 were treated with bromocriptine on top of heart failure medication. In none of them a bromocriptine-elated adverse event was reported [13]. Thus, we strongly favor the concept that in patients with PPCM low-dose bromocriptine therapy as given in our study (and also by many physicians as an individual treatment approach) is justified despite the concerns put forward in the EMA document. Based on the afore mentioned data the Study Group on Peripartum Cardiomyopathy of the Heart Failure Association of the European Society of Cardiology has requested the revision of the endorsement by the EMA and the permission for application of bromocriptine in PPCM patients under close medical supervision.

## Discussion

The results of this prospective, multicentre, randomized, open-label study will allow to determine whether bromocriptine on top of standard heart failure medication leads to improvement of LV function after 6 months in women with PPCM. In a very recent nationwide population-based study in the USA, the incidence and outcomes of PPCM were investigated in detail [3]. The authors demonstrated an increase of the incidence rate of PPCM, from 8.5 per 10,000 life births in 2011 to 11.8 per 10,000 life births in 2014. Importantly, the rate of maternal major adverse events defined as cardiac arrest, heart transplant, mechanical circulatory support, acute pulmonary edema, thromboembolism, or implantable cardioverter defibrillator/permanent pacemaker implantation increased from 11.7 % in 2004 to 15 % in 2011. Similar trends were observed for in-hospital mortality with an increase from 0.7 % in 2004 to 1.3 % in 2011. These data demonstrate that despite advances in medical care in Western societies during the last decade, the treatment concepts so far have failed to achieve improvements of the overall prognosis of PPCM patients. Therefore, with the rising incidence of PPCM and unaltered prognosis and lack of disease-specific therapeutics, there is an urgent need for novel therapeutic strategies [22]. This study is the first of this kind, and its results may contribute to the development of therapeutic strategies for the management of PPCM [3, 22].

The design of this study has been encouraged by a previous pilot study [12] and the findings of the non-randomized German PPCM registry [5] indicating beneficial effects of bromocriptine on LV function in PPCM. However, the current study is not fully comparable with the study be Sliwa et al. [12], which was placebo controlled as opposed to our study which compares low-dose short-term treatment of bromocriptine with prolonged bromocriptine treatment. This study design was based on ethical concerns about the continuation of lactation despite treatment of the patients with heart failure medication with potential side effects for the newborn, and because of the strong therapeutic effect of bromocriptine in the pilot study. Moreover, our ethic committee pointed out that stopping lactation without offering medical support is not allowed in Germany. The rationale for this treatment concept is based on experimental and clinical observations that a cleaved form of the nursing hormone prolactin, the 16 kDa prolactin, plays a causal role in the development of PPCM [9, 12]. This cleaved peptide exerts strong angiotoxic effects, in particular on the microvasculature, ultimately leading to cardiomyocyte cell death, and fibrosis. This hypothesis is supported by the evidence of increased serum levels of 16 kDa prolactin and augmented activity of the cleaving enzyme, cathepsin D [5, 12], at baseline in PPCM patients as compared to postpartal healthy women. Therefore, early pharmacological blockade of prolactin with bromocriptine may eliminate the detrimental effects of 16 kDa prolactin, prevent damage of the microvasculature, cell death, and replacement fibrosis, and thus, avoid adverse remodeling of the diseased myocardium and improve cardiac function and clinical condition of patients with acute onset of PPCM [9, 12]. Moreover, additional pleiotropic effects of bromocriptine are assumed to contribute to the healing process of the injured myocardium in PPCM. For example, proinflammatory pathways are known to be involved in the pathophysiology of PPCM [8, 23], which appear to be modulated upon treatment with bromocriptine [9, 23]. Further studies are warranted to identify precise mechanisms by which bromocriptine modulates the immunological responses that are activated in the development of PPCM. Other beneficial effects that have been attributed to bromocriptine are potential cytoprotective and antioxidative capabilities that could compensate for defective antioxidative mechanisms proposed as an important pathophysiological aspect in PPCM [8, 9]. Taken together, the cardioprotective effects of bromocriptine are broader than just effective prolactin blockade.

The basis of the dose and duration of bromocriptine therapy in the present trial were previous observations in animal models and case reports [11] which appeared to be efficient in the pilot bromocriptine study [12]. The change of LVEF was chosen as the primary endpoint as it is suitable for relatively small sample sizes of rare diseases such as PPCM.

Although blinding of the study is not possible due to ethical concerns, the data analysis and assessment of outcome is performed by blinded investigators which may partly compensate for the unavoidable open-label design.

To date, the observations obtained from this study indicate good safety and tolerability of bromocriptine in PPCM patients. So far, none of the patients had to prematurely terminate bromocriptine medication due to safety concerns. In particular, no bromocriptine-associated thrombotic complications were recorded. Only one patient experienced an adverse event in terms of temporary nausea.

In summary, this study addresses the potential of bromocriptine as add-on therapy in addition to standard heart failure therapy to improve LV function in acute PPCM. While initial results point to an adequate level of safety and tolerability, final follow-up evaluation of all planned patients need to be awaited to definitely determine the effect of bromocriptine on LV function in patients with acute PPCM. The results of this trial may have decisive impact on future management of PPCM patients.
